# Potential independent action of sigma receptor ligands through inhibition of the Kv2.1 channel

**DOI:** 10.18632/oncotarget.19581

**Published:** 2017-07-26

**Authors:** Xinying Liu, Yingmei Fu, Huan Yang, Timur Mavlyutov, Jun Li, Christopher R. McCurdy, Lian-Wang Guo, Bikash R. Pattnaik

**Affiliations:** ^1^ Departments of Pediatrics, University of Wisconsin School of Medicine and Public Health, Madison, WI, USA; ^2^ Department of Surgery, University of Wisconsin School of Medicine and Public Health, Madison, WI, USA; ^3^ Shanghai Key Laboratory of Psychotic Disorders, Shanghai Mental Health Center, Shanghai Jiao Tong University School of Medicine, Shanghai, PR China; ^4^ Department of Anesthesiology, University of Wisconsin School of Medicine and Public Health, Madison, WI, USA; ^5^ Department of Ophthalmology, The Third People's Hospital of Dalian, Dalian, PR China; ^6^ Department of Medicinal Chemistry, College of Pharmacy, University of Florida, Gainesville, FL, USA; ^7^ McPherson Eye Research Institute, University of Wisconsin, Madison, WI, USA; ^8^ Department of Surgery and Department of Physiology & Cell Biology, Davis Heart and Lung Research Institute, The Ohio State University, Columbus, OH, USA; ^9^ Department of Pediatrics, Department of Ophthalmology and Visual Sciences, University of Wisconsin School of Medicine and Public Health, Madison, WI, USA

**Keywords:** Kv2.1 channel, CRISPR/Cas9, sigma receptor ligands, patch-clamp, electroretinogram

## Abstract

The sigma-1 receptor (σ1-R) and sigma-2 receptor (σ2-R) are potential drug targets for treatment of cancer, pain, depression, retinal degeneration and other neuronal diseases. Previous reports show that sigma-1 receptor modulates the activities of multiple channels. We are interested in possible sigma receptor modulation of Kv2.1, a K^+^ channel abundant in retinal photoreceptors. We tested the effect of established sigma receptor ligands on Kv2.1 channels which were stably expressed in HEK293 cells. Surprisingly, σ1-R antagonists inhibited Kv2.1 currents in both wild type and σ1-R knockout HEK293 cells that we engineered using the CRISPR/Cas9 technology. Moreover, PB28, a σ1-R antagonist and also σ2-R agonist, inhibited Kv2.1 in σ1-R knockout cells, but this action was not blocked by the σ2-R antagonists that did not have an effect on Kv2.1. We also observed inhibition of electroretinogram by PB28 in wild type as well as σ1-R knockout mice. Thus, the results in this study indicate that the Kv2.1-inhibiting function of the sigma ligands is not sigma receptor dependent, suggesting a direct effect of these ligands on the Kv2.1 channel.

## INTRODUCTION

Sigma receptors are classified into the sigma-1 (σ1-R) and sigma-2 receptor (σ2-R) subtypes based on their distinct as well as shared ligand binding characteristics [1]. The σ1-R is a molecular chaperone primarily residing in the endoplasmic reticulum (ER) membrane with an ability to translocate to the plasma membrane [2]. The σ2-R has not been cloned [3]. Both σ1-R and σ2-R are ubiquitously distributed in mammalian tissues with high abundance in the central nervous system including the retina, implicating their functions in key physiological and pathophysiological processes [4]. Indeed, growing evidence indicates an important role of σ1-R in neurodegenerative diseases [5, 6]; pharmacological activation of σ1-R rescues degenerate cones in the rd10 mouse model of retinitis pigmentosa [7].

σ1-R is considered as a pluripotent modulator involved in a plethora of cellular signaling activities [8]. A great majority of σ1-R (and all σ2-R) modulations were identified via pharmacology using σ-R agonists and antagonists, many of which are known to bind other targets too [9]. The best known σ1-R function probably is its modulations of voltage-gated Ca^2+^ [10–13], Na^+^ [14–16] and K^+^ channels [17–19], via direct or indirect interactions [20]. σ1-R inhibits the activity of several K^+^ channels, including Kv1.2, Kv1.3, and Kv1.4 [18, 19, 21]; its interactions with these proteins were confirmed by co-immunoprecipitation experiments [19, 21].

Kv2.1 channels play an important role in neuronal systems including the neural retina [22]. The diverse functions of Kv2.1 include regulations of neuronal excitability and neural transmitter release. Recently, a prominent pro-apoptotic role of Kv2.1 in neurons has been recognized, and inhibition of Kv2.1 has shown neuroprotective effects [23]. As such, Kv2.1 is proposed to be a potential novel therapeutic target for treating neurodegenerative diseases [24] [23]. Interestingly, in our recent study using an Amyotrophic Lateral Sclerosis (ALS) mouse model, σ1-R in motoneuron subsurface ER cisternae was found in juxtaposition with Kv2.1 in the plasma membrane, implicating possible σ1-R regulation of Kv2.1 activity through direct or indirect interactions [25]. In light of the reported opposite roles of σ1-R and Kv2.1 in neuronal cell survival as well as their subcellular proximity in motoneurons, we hypothesized that activating σ1-R with an agonist would have an inhibitory effect on Kv2.1 activity; this may partially account for the neuroprotective role of σ1-R in degenerate retinas.

Therefore, in this study we set out to test the effect of established σ1-R agonists and antagonists on Kv2.1 current in a Kv2.1 stable-expression human cell line. We were able to engineer the Kv2.1-expressing cell line to knock out σ1-R, using the CRISPR/Cas9 genome-editing technology. This σ1-R knockout cell line provided a powerful tool to delineate whether a σ-R ligand effect on Kv2.1 is σ1-R-specific. To our surprise, while σ1-R agonist PRE084 showed no effect, σ1-R antagonists BD1047 and NE100 inhibited Kv2.1 current in both σ1-R wild type (WT) and σ1-R knockout (KO) cells; while σ2-R agonist PB28 potently inhibited Kv2.1, this action was not blocked by a highly σ2-R-selective novel antagonist (CM398) [3]. These results uncovered a paradoxical σ-R-independent inhibition of Kv2.1 by selective σ-R ligands.

## RESULTS

### σ1-R antagonists BD1047 and NE100 but not agonist PRE084 inhibit Kv2.1 current in Kv2.1-overexpressing HEK293 cells

**Figure 1:**
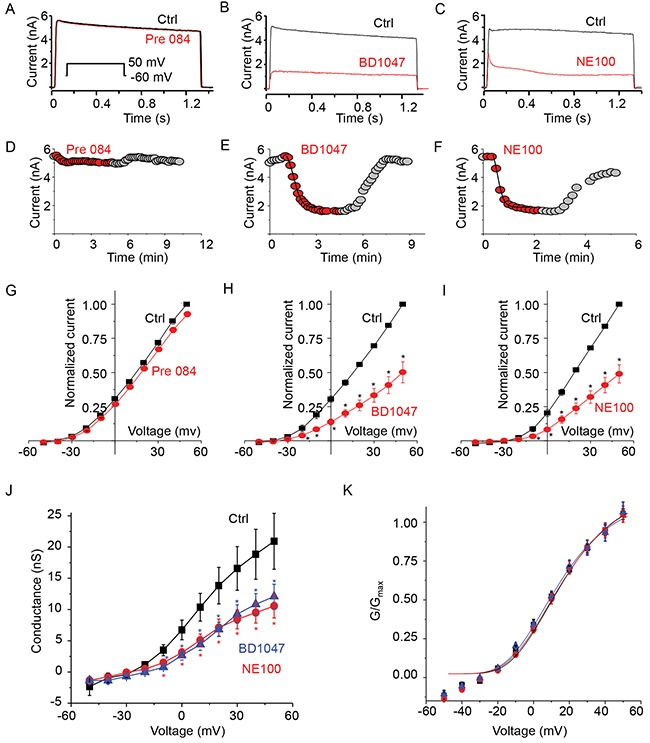
Only s1-R antagonists inhibit Kv2.1 channel current **(A)** Representative current traces from a cell expressing Kv2.1 channel before (HR; black trace) and during treatment with σ1-R receptor agonist PRE084 (10 μM, red trace). Inset voltage pulse to 50 mV from a holding potential of -60 mV. **(B)** Representative Kv2.1 channel current traces from a cell treated with σ1-R antagonist BD1047 (50 μM, red trace) compared to total current (black trace). **(C)** Comparison of Kv2.1 current traces before (HR, black trace) and during treatment with σ1-R antagonist NE100 (50 μM, red trace). **(D)** Time course of current amplitude at +60 mV. Recordings during Pre84 treatment is represented as red filled circles. **(E)** Filled circles (red) showing current amplitude time course during treatment of Kv2.1 expressing cells with BD1047. **(F)** Kv2.1 outward current amplitude as in D in presence of NE100 (50 μM, red filled circles). Both in E and F the solid line represents single exponential curve fit. **(G)** Average Kv2.1 whole-cell normalized current-voltage plot obtained in control solution (black squares) and during application of Pre084. **(H)** Comparison of average normalized current-voltage curve in presence of control bath solution and BD1047. **(I)** Average normalized current-voltage curve determined in control solution (black squares) and NE100 (red circles). **(J)** The G-V relationships of the control (solid black square), and in presence of BD1047 (blue triangle) or NE100 (red circle) are illustrated. **(K)** Normalized G-V curves as in J were fitted with the Boltzmann function. Data points in G-K are mean ± SEM (n=5). * P < 0.05 compared to control.

To test our hypothesis that activation of σ1-R inhibits the Kv2.1 channel, we used a HEK293 cell line stably overexpressing the Kv2.1 protein, as HEK293 cells are characteristic of extremely low intrinsic channel activities and to our advantage, these cells express sigma receptors [26]. Kv2.1 current was measured after the application of highly σ1-R-selective agonist PRE084 [27]. We found that treatment of cells with 10 μM of PRE084 had no effect on Kv2.1 current amplitudes measured as a voltage pulse to +50 mV from a holding potential of -60 mV (Figure 1A). However, upon treatment of Kv2.1-expressing cells with a commonly used σ1-R antagonist BD1047 (50 μM) [27], we noticed that the current amplitude was severely reduced (Figure 1B). We then used another established high-affinity σ1-R antagonist, NE100 (50 μM) [9, 27], to verify the BD1047-induced effect. We found that NE100 also reduced current amplitude (Figure 1C).

Time course of drug effect on Kv2.1 current at +50 mV is shown in Figure 1D, 1E, and 1F. Five minute application of Pre84, did not show any rundown of the outward current (Figure 1D). The reduction in the current during the application of BD1047 was quick (inhibition time constant 34.45 sec) and reversible (Figure 1E). NE100 inhibition of Kv2.1 current was rapid (inhibition time constant of 21 sec) and reversible (Figure 1F) during the six minute experiment. Average response of the three drugs on Kv2.1 is presented as current-voltage (I-V) plot comparison. Pre84 did not change Kv2.1 I-V plot (Figure 1G). In response to a +50 mV voltage pulse, Kv2.1 current density measured 152 ± 22 pA/pF that was only reduced to 143 ± 21 pA/pF (P < 0.5) after treatment with PRE084. The I-V plot showed that BD1047 inhibited outward current only (Figure 1H). Average current density at +50 mV was reduced from 135 ± 9 pA/pF to 67 ± 15 pA/pF (60 % inhibition, P < 0.05). Similarly, we observed a significant reduction in the outward current by NE100 on comparing the I-V relationship (Figure 1I). Average current density measured at + 50 mV were 149 ± 17 pA/pF in control solution compared to 77 ± 13 pA/pF in the presence of NE100 (P < 0.05) indicating 50% inhibition of Kv2.1 channel current.

We wondered if these inhibitors had any role in voltage dependent activation or gating of Kv2.1 channel. The plot of steady-state voltage activation showed a clear saturation within the membrane voltage range and an inhibition of outward conductance between -20 and -60 mV by both BD1047 and NE100 (Figure 1J). The half-activation voltage of control Kv2.1 channel (without ligand treatment) was 6 ± 0.38 mV. This was not significantly different from 4 ± 1.75 mV for BD1047 and 6 ± 1.05 mV for NE100 treatment (P < 0.5). There was no change either in slope conductance between the three conditions (Figure 1K). Taken together, our results conclude that the two σ1-R antagonists but not agonist PRE084 are able to inhibit the Kv2.1 channel.

### Knockout of σ1-R does not affect the Kv2.1 inhibitory effect of BD1047 and NE100

**Figure 2:**
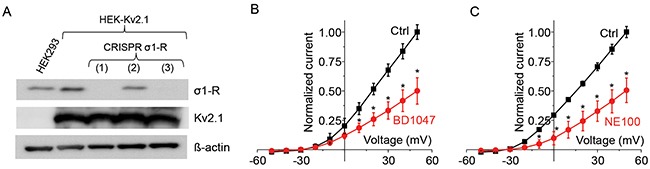
Antagonists inhibits Kv2.1 current after CRISPR/Cas9 mediated σ1-R receptor knockout **(A)** Western blot confirmed protein levels for σ1-R, Kv2.1, and ß–actin. Lane 1 represents HEK293 cells and lanes 2-5 represent HEK-Kv2.1 cells. Three CRISPR/Cas9 mediated σ1-R KO cell samples are marked as 1, 2, and 3. **(B)** Normalized current-voltage curve before (black squares) and after (red circles) treatment of cells, as in lane 3(A), with BD1047. **(C)** Normalized current-voltage curve for no drug control and with 50 M NE100. Data points in B and C are mean ± SE of the mean from at least 5 independent recordings and *P<0.05.

To investigate whether this Kv2.1 inhibition by σ1-R antagonists was truly mediated by σ1-R, we used the CRISPR/Cas9 technology to specifically knock out the σ1-R protein in Kv2.1-overexpressing HEK293 cells. As indicated by Western blotting (Figure 2A), σ1-R knockout was complete when using gRNAs #1 and #3 but not #2, and Kv2.1 protein levels were not altered after σ1-R depletion. We chose gRNA#1 to produce σ1-R KO cells for experiments throughout this study.

We then tested the effect of σ1-R antagonists on Kv2.1 current in σ1-R KO (and Kv2.1-overexpressing) HEK293 cells. As illustrated in Figure 2B, BD1047 inhibited outward current between -20 to +50 mV. In response to BD1047, outward current at +50 mV membrane potential was reduced from 135 ± 9 pA/pF to 67 ± 15 pA/pF (P < 0.05, n = 5). Similarly, σ1-R antagonist NE100 inhibited Kv2.1 outward current between -30 to +50 mV as shown in Figure 2C. In 6 cells, NE100 inhibited 50% of Kv2.1 current at +50 mV membrane potential, which was reduced from 118 ± 9 to 56 ± 12 pA/pF (P < 0.05, n = 6). Similar to the observation with σ1-R WT cells (Figure 1), inhibition of Kv2.1 current in σ1-R KO cells occurred within seconds after application of ligands (not shown). These results suggest that the inhibition of Kv2.1 current by σ1-R antagonists NE100 and BD1047 is σ1-R independent.

### σ2-R agonist PB28 potently inhibits Kv2.1 current in both σ1-R WT and σ1-R KO cells

**Figure 3:**
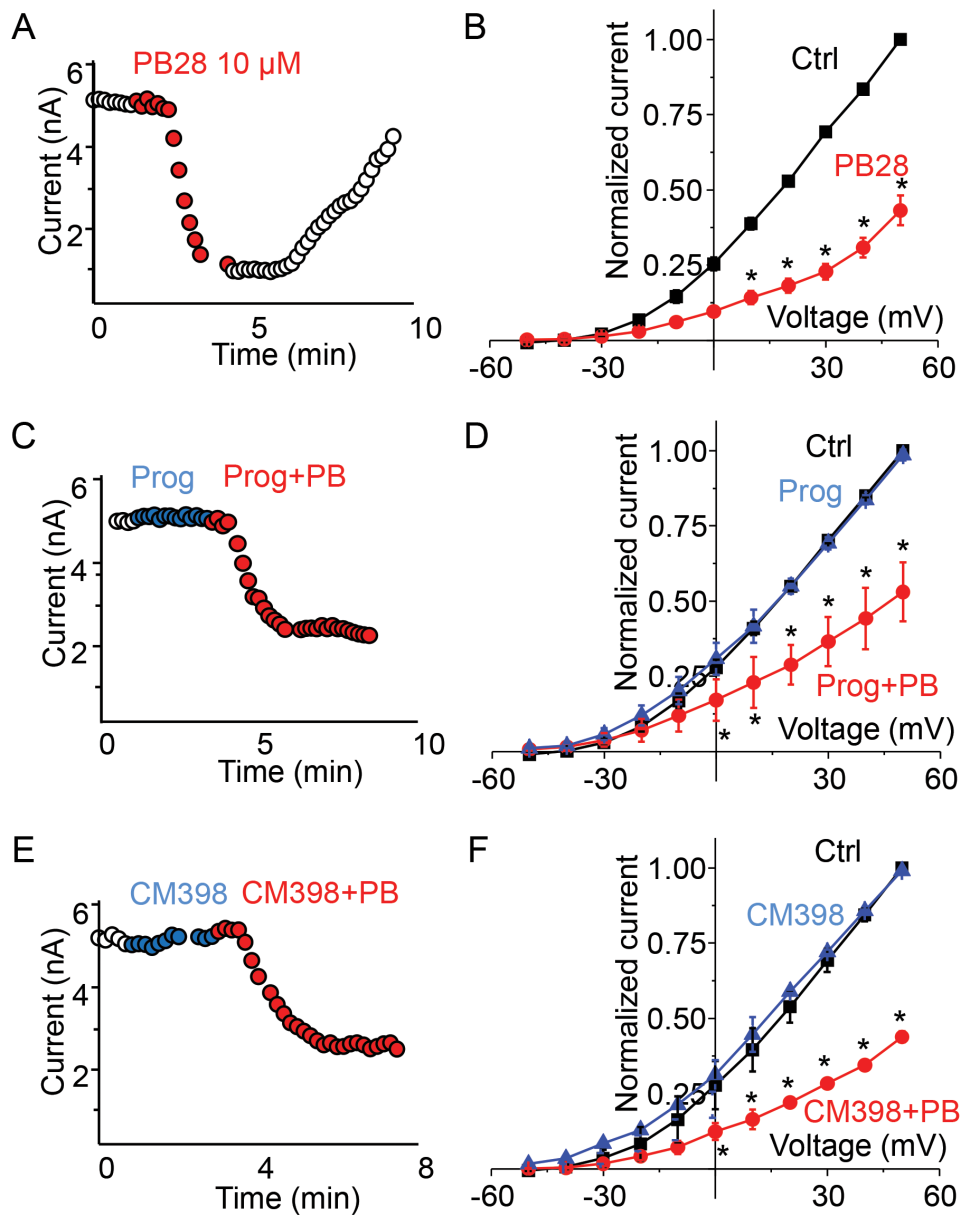
σ2-R agonist PB28 but not antagonists inhibits Kv2.1 current in σ1-R knockout HEK 293 cells expressing Kv2.1 channel **(A)** Representative cell showing current amplitude time course over 10 minutes with duration of PB28 application shown as red circles. **(B)** A plot of average normalized current-voltage curve in control (black trace) and after PB28 treatment (red trace). **(C)** Time course of current amplitude for a cell during the treatment of progesterone 20 μM (blue circles) or during the treatment of progesterone 10 μM and PB28 10 μM (red circles). **(D)** Average normalized current-voltage plot showing the effect of progesterone (blue trace) and progesterone plus PB28 (red trace) on Kv2.1 current. **(E)** In a representative cell current amplitude time course during the application of CM398 20 μM alone (blue circle) or CM398 10 μM plus PB28 10 μM (red circle). **(F)** Average plot of normalized current-voltage plot comparing Kv2.1 channel current responses to CM398 (blue trace) of CM398 plus PB28 (red trace) treatment.

Although BD1047 and NE100 are deemed σ1-R selective ligands, they also bind σ2-R albeit with ~40 fold lower affinities [9]. Thus, an interesting question arose as to whether the observed inhibition of the Kv2.1 channel was actually mediated by σ2-R. Our σ1-R KO HEK293 cells provided a unique opportunity to address the role of σ2-R because the binding of a σ2-R ligand to σ1-R was ruled out. We opted to use a well-established high-affinity σ2-R agonist (PB28) for investigation [28]. PB28 is also a high-affinity σ1-R antagonist thus ideally representing σ-R ligands shared by the σ1-R and σ2-R subtypes [28]. In Figure 3, we show the effect of PB28 on Kv2.1 current in the σ1-R KO HEK293 cells. We noticed a progressive decrease in outward current following PB28 application, and the inhibition of Kv2.1 current was PB28 concentration dependent (data not shown). Since 10 μM PB28 (Figure 3A, red circle) nearly abolished Kv2.1 outward current as measured at +50 mV, we chose this concentration for later experiments. Although PB28 binds to σ1-R and σ2-R with a Kd (or Ki) in the sub-nanomolar range, the affinities were determined with membranes isolated from lysed cells. In studies using intact cells, PB28 concentrations ranging from 5-50 μM have been commonly used [29]. The time course of Kv2.1 current inhibition by 10 μM PB28 is shown in Figure 3A. The inhibition was quick (inhibition time constant of 22 sec) and completely reversible. The average plot of the I-V curve (Figure 3B) also showed outward current inhibition between -30 to +50 mV. The Kv2.1 current of 132 ± 3 pA/pF measured at +50 mV membrane potential was reduced to 47 ± 10 pA/pF in the presence of 10 μM PB28, indicating a 65% inhibition of current (P < 0.005, n = 6).

### Inhibition of Kv2.1 by σ2-R agonist PB28 is not blocked by σ2-R antagonists

We then measured the effect of two σ2-R antagonists, progesterone and CM398, on Kv2.1 current in σ1-R KO HEK293 cells. As illustrated in Figure 3C, progesterone at 20 μM had no effect on Kv2.1 current amplitude during more than 2 minutes of application (Figure 3C blue circles). Addition of PB28 (10 μM) together with progesterone (10 μM) still rapidly inhibited Kv2.1 current (Figure 3C red circles). Figure 3D further summarizes our findings that progesterone had no effect on Kv2.1 current voltage curve (Figure 3D, blue triangle), and PB28 inhibited outward current between -30 to +50 mV regardless of the presence of progesterone (Figure 3D, red circles). Current amplitude at +50 mV membrane potential measured to be 133 ± 11 pA/pF for control (no ligand), 132 ± 12 pA/pF (P < 0.5, n = 4) for progesterone, and 61 ± 8 pA/pF (P < 0.005, n = 4) for PB28 plus progesterone treatment. A novel highly σ2-R-selective antagonist (>1000 fold over σ1-R), CM398 (20 μM) [3], had no effect on Kv2.1 current either (Figure 3E, blue circle) during approximately 2 minutes of drug application. Subsequent addition of PB28 (10 μM) and CM398 (10 μM) reduced the current amplitude (Figure 3E, red circles). On an average, current-voltage plot showed no effect of CM398 on PB28 inhibition of Kv2.1 outward current (Figure 3F). At +50 mV the amplitudes measured 143 ± 7 pA/pF for control, 144 ± 9 pA/pF for CM398 (P < 0.5, n = 3), and 55 ± 11 for PB28 and CM398 (P < 0.05, n = 3). These results support an assertion that both σ2-R antagonists progesterone and CM398 do not block the Kv2.1-inhibiting action of σ2-R agonist PB28, indicative of a σ2-R-independent effect.

**Figure 4:**
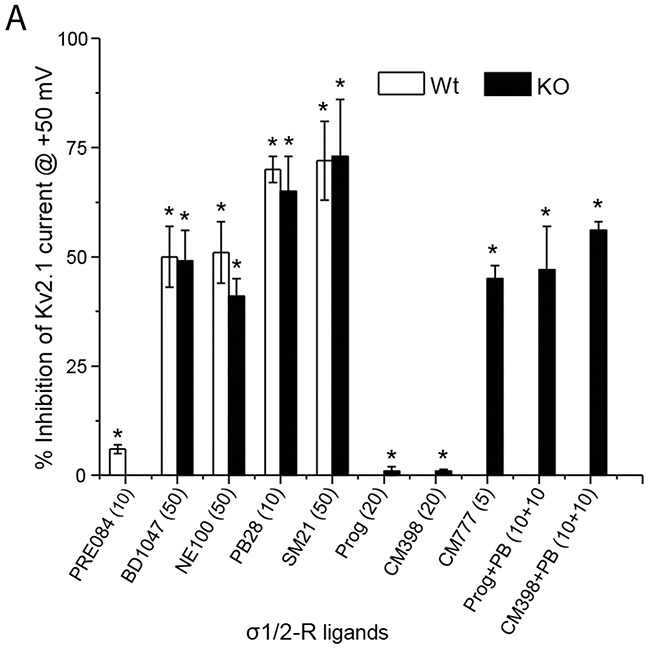

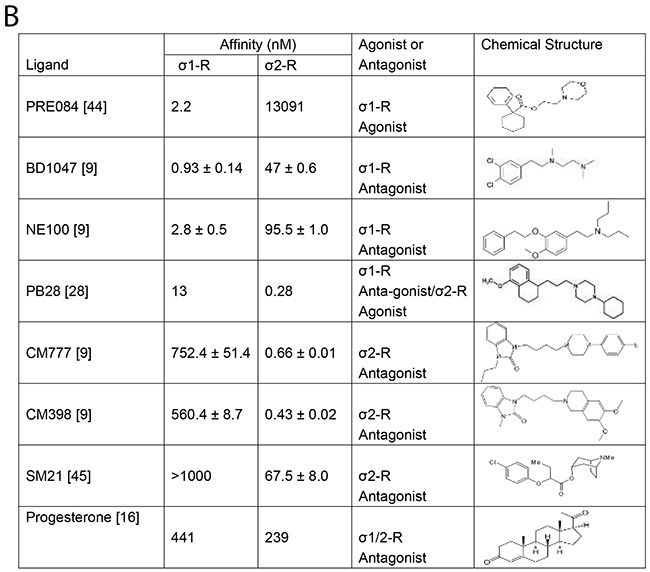
Summary of σ1 and 2-R ligands inhibition of Kv2.1 current **(A)** Bar graph summary of average % inhibition of Kv2.1 channel in HEK293 cells (open bar) or after σ1-R knockout (black bar) in HEK 293 cells in presence of various σ1- and σ2-R ligands. Data is mean ± SEM (n = 4 at least) and *P < 0.05. Concentrations of the ligands are presented in the parentheses under the bar graph. **(B)** σ1-R and σ2-R binding affinities, functions, and chemical structures of σ1-R ligands.

The results from our experiments using various σ-R ligands are summarized in Figure 4. While σ1-R agonist PRE084 did not inhibit the Kv2.1 channel, σ1-R antagonists BD1047, NE100, and PB28 each inhibited Kv2.1 current to similar degrees in σ1-R WT and KO cells. σ2-R agonists PB28 and SM21 potently inhibited Kv2.1 in both WT and KO cells, but the PB28 inhibitory effect could not be blocked by σ2-R antagonists progesterone and CM398. Whereas progesterone and CM398 did not show an inhibitory effect, another σ2-R antagonist, CM777 [3], strongly inhibited Kv2.1 activity. On the other hand, DTG as a classic sigma receptor ligand that binds to both σ1-R and σ2-R (affinities: ~50 nM) did not inhibit Kv2.1 even at 50 μM (data not shown). These results together indicate that those Kv2.1-inhibiting σ-R ligands acted independently of σ1-R and σ2-R.

### PB28 reduces ERG a-wave and b-wave amplitudes in σ1-R WT and KO cells

**Figure 5:**
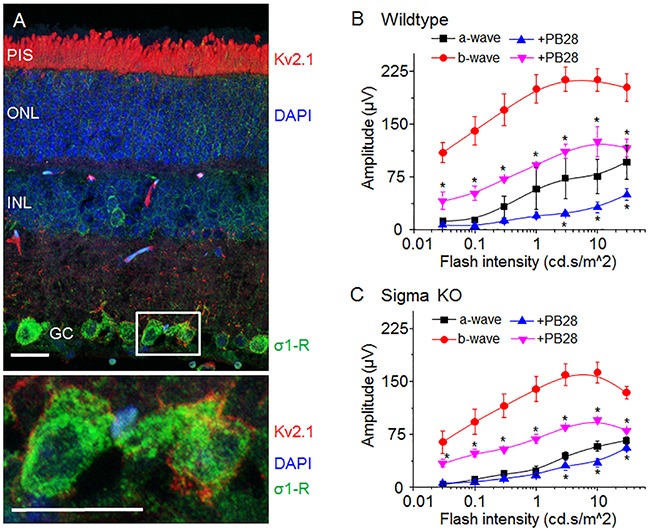
σ-R ligand PB28 attenuate mouse ERG possibly through Kv2.1 inhibition **(A)** Micrograph of mouse retina showing localization of Kv2.1 (red) and σ1-R (green). Nuclear layer is shown as blue DAPI staining. In the lower panel two enlarged ganglion cells marked with a white box showing Kv2.1 (red) and σ1-R (green) immunostaining. Scale bar 15 μM. **(B)** Average response of a- and b-wave amplitude in relation to light flash intensity. Vehicle injected eye response is shown as black (a-wave) and red (b-wave) traces. PB28 injected eye is shown as blue (a-wave) and purple (b-wave) traces. **(C)** Average ERG a- and b-wave responses from σ1-R knock out mice after saline or PB28 injection. Color representation as in B. Data is represented as mean ± SEM from at least 5 observations for each point and *P < 0.05 defines significance compared to control.

The Kv2.1 channel is highly expressed in retinal photoreceptor cells although its functions in mammalian retinas are not well understood [22, 30]. Consistently, our immunostaining data show that Kv2.1 is predominantly localized in the photoreceptor inner segment (Figure 5A, red staining) with minor amounts in other retinal layers. In contrast, while present in all retinal layers, σ1-R is more abundant in the ganglion cell layer (Figure 5A, green staining), as also observed previously in our and other's studies [31, 32]. As shown on the magnified images of two ganglion cells (Figure 5A lower panel), although Kv2.1 in the plasma membrane was in proximity to σ1-R in the ER, there was no appreciable overlap of immunostained Kv2.1 and σ1-R.

Because of the abundance of the Kv2.1 channel in photoreceptors in the mouse retina and its inhibition by PB28 *in vitro*, we explored a possible impact of PB28 on electroretinogram (ERG) *in vivo*. We first determined the effect of PB28 on ERG in WT mice, via intravitreal injection of PB28 into one eye and vehicle control (equal amount of DMSO in PBS) into the contralateral eye. Figure 5B shows scotopic ERG amplitudes of a- and b-waves after administration of PB28 or vehicle, which were elevated in response to increasing light flash stimulus, indicating retinal responses induced specifically by light. Interestingly, compared to vehicle control (a-wave, black; b-wave, red; Figure 5B), PB28 substantially reduced average a-wave (Figure 5B, blue) and b-wave (Figure 5B, purple) amplitudes by ~50%.

Considering reported σ1-R modulations of various channels; Ca^2+^ [10–13], Na^+^ [14–16] and K^+^ channels [17–19], some present in the retina, we performed the same ERG experiments also in σ1-R KO mice to assess whether σ1-R is possibly involved in the PB28-induced ERG inhibition. As indicated in Figure 5C, PB28 also inhibited a-waves in σ1-R KO mice (Figure 5C, blue vs black) albeit to a less degree relative to an ~50% inhibition in WT mice (Figure 5B). This difference between σ1-R KO and WT suggests a partial involvement of σ1-R in PB28-induced a-wave attenuation. However, like in WT mice, b-wave amplitudes were still substantially reduced by ~50% after PB28 injection in σ1-R KO mice (Figure 5C, purple vs red), demonstrating a strong non-σ1-R effect. In addition, while the basal levels (no PB28) of a-waves were slightly lower in σ1-R KO versus WT mice, b-waves were markedly smaller in σ1-R KO mice (highest at 150 μV *vs* 220 μV compared to WT, in Figure 5C and 5B, respectively, red curves).

These results suggest that PB28 inhibition of mouse ERG *in vivo* is partly σ1-R associated, and also by mechanisms independent of σ1-R, which possibly involves Kv2.1.

## DISCUSSION

We made an unexpected finding that a defined group of σ-R-selective ligands potently inhibit Kv2.1 currents paradoxically in an σ-R-independent manner. Both σ-Rs and Kv2.1 are broadly distributed with diverse functions, especially in neuronal systems. Prompted by known σ1-R /channel interactions [20] and σ1-R juxtaposition with Kv2.1 [25], we initially sought to test a possible σ1-R modulation of Kv2.1 activity. Surprisingly, our data revealed that a few high-affinity σ-R ligands inhibited Kv2.1 regardless of σ-R activity. Although some σ-R ligands have been reported to bind other proteins as well [9], little is known about ion channels as alternative targets of highly σ-R-selective ligands. Our findings may thus open new perspectives in pharmacological manipulations involving σ-Rs and/or the Kv2.1 channel, both emerging intervention targets.

As revealed by a series of unexpected results, the observed Kv2.1-inhibiting effect of σ-R ligands was independent of both σ1-R and σ2-R. The first surprise was that σ1-R agonist PRE084 had no effect on Kv2.1 currents, in contrast to widely reported σ1-R modulations of various channels, including Kv members [20]. Instead, we found that σ1-R antagonists BD1047 and NE100 strongly inhibited Kv2.1 activity. Surprisingly, in σ1-R KO cells they inhibited Kv2.1 current to the same extent as in σ1-R WT cells. This result precludes a functional involvement of σ1-R. Further; we found that high-affinity σ2-R agonist (and also σ1-R antagonist) PB28 abolished Kv2.1 function in σ1-R WT as well as σ1-R KO cells, implicating a σ2-R-related mechanism. However, neither progesterone nor CM398, both σ2-R antagonists [9], were able to block the PB28 inhibition of Kv2.1 current, indicating that the PB28 action is a non-σ2-R effect. On the other hand, other two structurally distinct σ2-R antagonists (CM777 and SM21) showed Kv2.1-inhibitory potency [9]. However, the result that high-affinity σ2-R agonist PB28 and antagonist CM777 both potently inhibit Kv2.1 strongly argues against a σ2-R-specific effect of these two σ2-R ligands. Moreover, DTG as both a σ1-R and a σ2-R ligand without known off-targets did not inhibit Kv2.1 at 50 μM (data not shown). Therefore, our results are compelling in supporting a σ-R-independent Kv2.1-inhibiting function of the previously deemed “σ-R-selective” ligands.

An alternative explanation would be that these Kv2.1-inhibiting ligands inhibit Kv2.1 indirectly via a σR/Kv2.1 interaction, but the σR-mediated effect is “masked” by overexpressed Kv2.1 protein. If a σR/Kv2.1 interaction were true, overexpression of Kv2.1 would greatly increase σR/Kv2.1 contacts, and a difference made by σ1-R depletion would be amplified. However, we did not observe a difference in σ1-R ligand-induced Kv2.1 inhibitory effects between σ1-R WT and σ1-R KO cells, thus a functional σ1R/Kv2.1 association was ruled out. In support of the lack of σ1-R/Kv2.1 protein-protein interaction, in a recent study, σ1-R co-immuno-precipitated with Kv1.2 but not Kv2.1 in the mouse brain tissue [21]. Moreover, our immunostaining images did not show evident co-localization between Kv2.1, a plasma membrane protein, and σ1-R, an ER resident [2]. Another scenario is that σ-R ligands bind to other ion channels (e.g., Ca^2+^, Na^+^) which indirectly influence Kv2.1 current. Although we cannot rule out this possibility definitively, inhibition of Kv2.1 current occurred rapidly (within 40s after ligand application), which may be most reasonably explained by ligand binding directly to the Kv2.1 protein. Moreover, in support of a Kv2.1-selective effect of the σ-R ligands, we used a Kv2.1 stable-overexpression HEK293 cell line, which features extremely low abundance of other ion channels [33]. Of note, a histamine- and serotonin-receptor antagonist, cyproheptadine, was recently shown to bind σ1-R and enhance outward K^+^ current mediated by the Kv2.1 α subunit [34]. Since cyproheptadine differs drastically from the Kv2.1-inhibiting ligands studied here, it is not clear whether it interacts with Kv2.1. Nevertheless, to prove or disprove direct binding of σ-R ligands to Kv2.1, it requires crosslinking a labeled σ-R ligand to Kv2.1 or ligand binding assay using purified functional Kv2.1 protein, which warrants future investigations.

Kv2.1 is a delayed rectifier-type potassium channel with diverse functions, including regulations of neuronal excitability and transmitter release, insulin secretion, and heart rate [35]. Because of a pro-apoptotic role in neurons and beta cells [23], Kv2.1 has recently become an attractive anti-neurodegenerative and anti-diabetic target [24]. While various agents have been found to inhibit Kv2.1 activity, reports of high-affinity small molecule Kv2.1 inhibitors remain scarce [24], hampering Kv2.1-modulatory therapeutic development. In this regard, our finding of novel potent Kv2.1 inhibitors is of timely importance. There has not been a reported pharmacophore of Kv2.1 inhibitors. The chemical structures of the known Kv2.1 inhibitor compounds vary drastically; e.g., donepezil, galantamine, RY796, and isoliquiritigenin [24]. It is intriguing that donepezil has been used as both a Kv2.1 inhibitor [23] and a σ1-R ligand [9]. As any ligand would bind off-targets at high concentrations, Kv2.1 may represent an off-target for σ-R ligands. Since a structurally similar subgroup of σ-R ligand, but not all of them, inhibited Kv2.1 to various extent we speculate that there is a direct interaction between sigma ligands and Kv2.1 protein. It is interesting to note the similarity between the structures of PB28 and CM777, the two most potent Kv2.1-inhibitory σ-R ligands characterized herein (Figure 4B). While each is composed of two ring structures connected by an alkyl chain, both contain a piperazine group. Significantly, if the cyclohexylpiperazine group is substituted by an isoquinolin group (in CM398), the Kv2.1 inhibitory function is abolished. The structures of other three Kv2.1-inhibiting σ-R ligands (BD1047, NE100, and SM21) differ substantially from CM777 and PB28 (Figure 4B), and their inhibitory potency is ~10 fold lower with an estimated IC50 at ~50 μM. Therefore, the structure-and-function information obtained here is useful for future development of more Kv2.1-selective therapeutic inhibitors. Given the fact that hundreds of compounds have been found to bind σ-Rs with some in clinical use or trials [9], σ-R ligands constitute a rich repertoire for discovering novel Kv2.1 inhibitors.

Another interesting observation is the inhibitory effect of PB28 on photoreceptor electrophysiology measured by ERG. Compared to studies in the brain and other tissues, Kv2.1 is much less investigated in the retina (total 8-10 PubMed publications). The exceptionally high expression of Kv2.1 in mouse photoreceptor inner segments implicates functional importance. However, in photoreceptors Kv2.1 co-assembles with Kv8.2 channel to mediate K^+^ efflux, and may thus facilitate strong hyperpolarization of photoreceptors [22]. This is also evidenced by the fact that the co-assembled channel generates unique biophysical characteristics identical to photoreceptor *I*_K_ [36]. In our *in vitro* study, inhibition of the Kv2.1 channel by σ-R ligands did not alter channel voltage-dependent activation or kinetics, hence we infer that these agents probably directly inhibit the function of a Kv2.1/Kv8.2 hetero-multimeric channel. Our mouse ERG recording showed severely reduced a-wave and also b-wave responses following intravitreal injection of PB28. This is consistent with the contribution of Kv2.1 channel to ERG a-waves due to the hyperpolarization of photoreceptors.

While a protective role of σ1-R for retinal neurons has been strongly advocated by a number of genetic and pharmacological studies [6, 7], its influence on photo-transduction remains underexplored [7, 32]. The role of σ2-R in the retina is not known. Here we found that compared to WT mice, PB28 inhibition of ERG a-waves were attenuated to some degree due to σ1-R knockout, suggesting a specific role of σ1R in retinal photoresponse. This is not surprising in light of the reported σ1-R interactions with various channels that are also present in retinal photoreceptors and secondary neurons [20]. Interestingly, a good portion of PB28 inhibition of ERG (especially b-waves) persisted in σ1-R KO mice, indicating a non-σ1-R effect. Although we cannot identify which targets were responsible for this effect, Kv2.1 may have at least partially mediated the PB28-induced ERG inhibition considering the great abundance of Kv2.1 in photoreceptors and potent PB28 inhibition of Kv2.1 activity measured *in vitro*. Future experiments using PB28 combined with guangitoxin-1E, a known Kv2.1 inhibitor, would help answer this question.

## MATERIALS AND METHODS

### Cell line and reagents

Human HEK293 cells were purchased from American Type Culture Collection (ATCC, Manassas, VA). HEK293 cells with stable expression on Kv2.1 channel was obtained from Durga Mohapatra. Cells were maintained in a complete growth medium (DMEM high glucose + 10% heat inactivated Fetal Bovine Serum + 1X Penicillin and Streptomycin + 1X Glutamax all from Lifetechnology, USA). For selection of Kv2.1 stable cells, 400 μg/ml of G418 was used. Cells were cultured up to 70 - 90% confluency in a 5% CO_2_ incubator at 37°C and 95% humidity. Cells were regularly sub-cultured to avoid over confluency.

Reagents: Puromycin dihydrochloride, Pre084 hydrochloride, BD1047 dihydrobromide, and NE100 were purchased from Tocris. PB28 was from Sigma-Aldrich. CM398 and CM777 were synthesized by Dr. McCurdy's laboratory [3]. All other reagents were purchased from Sigma-Aldrich or Thermo-Fisher unless specifically stated.

### Knockout of σ1-R using the CRISPR/Cas9 genome editing technology

To generate a σ1-R knockout HEK293 human cell line, a CRISPR/Cas9 genome-editing approach was used, as described in our previous report with minor modifications [3]. Briefly, three CRISPR guide RNAs (gRNAs) targeting the human σ1-R gene (*SIGMAR1*) were chosen. Targeted sequences are 5’-GGCCTTCTCTCGTCTGATCG-3’, 5’-TGACCCA GGTCGTCTGGCTC-3’, and 5’-GTGGGCCGTGGGCCG GCGGT-3’. Cloning of gRNAs into lentiCRISPR v2 and lentivirus production were performed as reported [38]. The cells were transduced with lentivirus for 3 days, and genome-edited cells were selected with 1 μg/ml puromycin for 1 week, and σ1-R KO was then verified by Western blotting.

### Western blotting to confirm σ1-R knockout

Following CRISPR/Cas9 genome-editing, selected HEK293 cells were collected and cell lysates were solubilized in RIPA buffer. Proteins were separated on a 4–20% Mini-PROTEAN TGX precast gel (Bio-Rad, Hercules, CA) and transferred to a PVDF membrane. A blot was incubated with a primary antibody: rabbit anti-σ1-R (Santa Cruz Biotechnology, Dallas, TX, 1:200), mouse monoclonal anti-Kv2.1 (Clone K89/34, NeuroMab, CA, 1:1000), or mouse anti-β-actin (Sigma-Aldrich, USA, 1:10000), washed 3 times, and then incubated with an HRP-conjugated secondary antibody (goat anti-rabbit or mouse, Bio-Rad, 1:5000). Specific protein bands on the blot were visualized by applying enhanced chemiluminescence reagents (Pierce, Rockford, IL) and then recorded with a LAS-4000 Mini imager (GE, Piscataway, NJ).

### Animals

All animal procedures conform to the NIH Guide for the Care and Use of Laboratory Animals. Animal protocols (M02102/M05434) were approved by the Institutional Animal Care and Use Committee at the University of Wisconsin–Madison. Heterozygous σ1-R knockout mice, namely *Oprs1* mutant (+/−) B6;129S5-Sigmar1Gt(OST422756) Lex/Mmucd mouse litters on a C57BL/6J × 129s/SvEv mixed background, were purchased from the Mutant Mouse Regional Resource Center (#011750, MMRRC, UC Davis, CA, USA). The colony of homozygous σ1-R KO mice was then established by backcrossing to C57BL/6J mice for >7 generations [39]. Litter mates with homozygous alleles of the sigma-1 receptor gene (*Sigmar1*) were used as wild type control. σ1-R WT and σ1-R KO litter mates were genotyped as previously reported [25] before the use for experiments. Animals were maintained on a 4% fat diet (8604 M/R, Harkland Teklad, Madison, WI) and subjected to standard light cycles (12 h/12 h light/dark). Both male and female mice in the age range of postnatal days 40–50 were used in experiments. Animals were euthanized in a chamber gradually filled with CO_2_.

### Immunohistochemistry for detection of Kv2.1 and σ1-R on retinal sections

Mice were euthanized by CO_2_ asphyxiation followed by cervical dislocation. Retinal cryosections were prepared according to our published methods [31, 40]. Briefly, eyeballs were fixed overnight in 4% paraformaldehyde at 4 °C and then soaked in 30% sucrose (in PBS) overnight at 4 °C, and 10 μm sections were cut from the eyeballs frozen in the optimum cutting temperature (OCT) embedding medium.

Immunostaining was performed on retinal cryosections following our previously described method [41] with minor modifications. Briefly, retinal sections were permeabilized with 0.1% Triton X-100 in PBS for 20 min, blocked with 5% normal donkey serum (Jackson Immunoresearch Lab, MS) for 1 h at room temperature, and then incubated with a primary antibody overnight at 4°C. Sources of primary antibodies are the following: rabbit anti-σ1-R serum (in-house produced, 1:700) [25]; Mouse monoclonal anti-Kv2.1 (Clone K89/34, NeuroMab, CA, 1:300) [25]. After rinsing the section 3×, a secondary antibody (Alexa-488 conjugated donkey-anti-rabbit or Alexa-555-conjugated goat-anti-mouse) at 1 μg/ml was applied at room temperature for 1 h. Sections were then rinsed and counterstained with DAPI and cover-slipped. Fluorescence images were acquired under a 60× oil objective lens with a Nikon A1RS confocal microscope (Nikon Inc, Mellville, NY).

### Electrophysiology

Kv2.1 channel currents were recorded by whole-cell patch clamp at room temperature using an Axopatch 200B amplifier (Molecular Devices, Sunnyvale, CA) and the Digidata 1440A data acquisition system (Molecular Devices, Sunnyvale, CA). The data were low-pass filtered at 1 kHz, and digitized at 0.5 kHz. The cell-covered glass coverslips were placed at the bottom of a 13 mm chamber (Warner Instruments, MA) mounted on the fixed stage of a Nikon FN-1 microscope and continuously perfused using the HEPES Ringer's (HR) buffer containing (in mM): 135 NaCl, 5 KCl, 10 HEPES, 10 glucose, 1.8 CaCl_2_, and 1 MgCl_2_. The pH was adjusted to 7.4 using NaOH.

Patch pipettes were made from borosilicate glass (BF150-117-10, Sutter Instruments, Novato, CA) using a horizontal pipette puller (P-1000, Sutter Instruments, Novato, CA) and fire polished (MF-830, Narishige, Tokyo, Japan). The recording electrodes, when filled with (in mM) 30 KCl, 83 K-gluconate, 10 HEPES, 5.5 EGTA, 0.5 CaCl_2_, 4 Mg-ATP, and 0.1 GTP, and adjusted to pH 7.2 using KOH, measured a tip resistance between 3.5–4.5 MΩ. Whole cell capacitance and series resistance were monitored and compensated throughout the recording. Gravity perfusion system was used for HR perfusion and sigma receptor ligand application. The Kv2.1 current was monitored either using a linear voltage ramp from −50 mV to +50 mV or a voltage step series between −50 mV to +50 mV. In between recordings, the cell was held at −60 mV. All recordings were performed at room temperature. We used 1^st^ order exponential nonlinear curve fitting equation; for the time course data

$$y\;=\;A1\;*\;\exp\;\left(-\frac{x}{t1}\right)\;+\;y0$$

to determine inhibition time constant in Origin (Origin Lab, Northampton, MA). Whole cell data was acquired and analyzed using pClamp10 and all plots were generated in Origin. Conductance was measured from the current amplitude divided by K^+^ ion driving force; *G* = *I*/(*Vm* − *Ek*) where *G* is conductance, *I* is current, *Vm* is membrane potential, and *Ek* is Nernst potential for K^+^. When normalized with the maximal conductance (G/G_max_), the curves were fit with a Boltzmann nonlinear curve fit; half maximal voltage dependence is *V0.5* and slope is *S*.

$$G\;=\;Gmin\;+\;\frac{\left(Gmax\;-\;Gmin\right)}{1\;+\;\exp\left(\left(V0.5\;-\;Vm\right)/S\right)}$$

### Electroretinogram

The animal procedures were in compliance with the ARVO Statement for the Use of Animals in Ophthalmic and Vision Research. Details of ERG recording were described in our recent report [40]. Briefly, 40-day old σ1-R WT and σ1-R KO mice were anesthetized with ketamine/xylazine (80 and 10 mg/kg IP). Mice were dark-adapted overnight and ERG measurements were performed using standard ISEVC protocol using a HmsERG system (Ocuscience, NV) the next day. ERG data were analyzed using ERGView and plotted in Origin.

### Intravitreal injection of PB28

Intravitreal injection was performed as we previously reported [42, 43]. Mice were anesthetized with the ketamine/xylazine (80/10 mg/kg IP) cocktail. Proparacaine hydrochloride (0.5%; Alcon Laboratories, Inc., Fort Worth, TX) and ofloxacin ophthalmic solution (0.3%; Allergan Inc., Irvine, CA) were applied to the ocular surface before injection for topical anesthesia and infection prevention, respectively. To avoid injuries to the lens, a ~0.5 mm incision posterior to the temporal limbus was first made using a 27-gauge single-use needle (BD, Franklin Lakes, NJ), and then a 30-gauge blunt-end needle (10 mm length; Hamilton, Reno, NV) in a Hamilton 701RN syringe was inserted through the incision. The needle was advanced approximately 1.5 mm deep while avoiding the lens, angled toward the optic nerve until the needle tip was seen in the center of the vitreous; then, 2 μl of solution was injected. PBS control (containing equivalent amount of DMSO) was injected to the left eye and PB28 in PBS (20 μM) was injected to the right eye. To ensure that no infection would occur, bacitracin ophthalmic ointment (E. Fougera & Co., Melville, NY) was applied immediately after pulling out the needle. Injections were performed using a UMP3 ultra micro-injection system (World Precision Instruments, Sarasota, FL)

### Statistical analysis

All experiments were performed at least five times. Data was analyzed for significance using Student t-test. Data was interpreted as significant when p value measured less than 0.05.

## CONCLUSIONS

Using a human cell line specifically overexpressing Kv2.1 and with σ1R depleted via genome editing, we identified an σ-R-independent Kv2.1-inhibitory function of a defined group of ligands that were previously characterized as σ-R-selective. The significance of our finding can be several fold. First, these σ-R ligands may represent a novel set of sought-after Kv2.1 inhibitors, with PB28 and CM777 being the most potent. Second, comparison among PB28 and CM777 (Kv2.1 inhibitory) and CM398 (Kv2.1 non-inhibitory) confers new knowledge of structure-activity relationship highlighting the importance of the piperazine group. Third, as the σ2-R gene sequence is not known [3], σ2-R ligands provide the only approach for σ2-R-targeted imaging and therapeutic investigations [37]. Our findings here recommend considerations of possible off-targets of σ2-R ligands. Moreover, σ1-R and σ2-R are considered as potential targets for treating a variety disorders, including addiction and psychotic disorders, neurodegenerative diseases, pain, and cancers [9]. Kv2.1 inhibition has been proposed as an approach to treat neurodegenerative (e.g. Alzheimer’s) and diabetic diseases [24]. Thus, optimal therapeutic outcomes may be attainable by conciliating drug effects on σ-Rs and the Kv2.1 channel. All these perspectives warrant continued research to open new avenues to advance Kv2.1 and/or σ-R-targeted therapeutic development.
